# Image Matching Algorithm-Driven Multi-Beam Customized Meta-Device

**DOI:** 10.3390/ma19010004

**Published:** 2025-12-19

**Authors:** Xingshuo Cui, Dan Liu, Borui Wu, Huiyong Zeng, Yueyi Qin, Guangzheng Ren, Guangming Wang, Tong Cai

**Affiliations:** 1 Air and Missile Defense College, Air Force Engineering University, Xi’an 710051, China; cxsgfs521@sina.com (X.C.); qinyy010404@163.com (Y.Q.); guangzheng_ren@163.com (G.R.); wgming01@sina.com (G.W.); 2 State Key Laboratory of Modern Optical Instrumentation, The Electromagnetics Academy Zhejiang University, Hangzhou 310027, China; 12231114@zju.edu.cn; 3 Air Traffic Control and Navigation School, Air Force Engineering University, Xi’an 710051, China

**Keywords:** metasurface, image matching algorithm, multi-beam, optimization design, amplitude-phase modulation

## Abstract

Achieving the multi-beam directional manipulation of electromagnetic waves and energy management of each beam has important application value in fields such as satellite communications. Existing methods for realizing multi-beam formation and energy distribution using metasurfaces have problems such as slow optimization speeds and low design accuracies. To break through this design bottleneck, a new design paradigm is proposed that introduces a 2D image matching algorithm into the design process of metasurfaces for multi-beam energy management. By using 2D grayscale images to characterize the far-field patterns generated by the metasurface array and the expected multi-beams, a wavefront characterization and matching optimization model driven by 2D grayscale images is established, ultimately achieving the customized design of beam position, intensity, and sidelobe constraints. The fully customized beam metasurface constructed using this method generates multiple *y*-polarized reflected directional beams under an *x*-polarized wave incidence, and the beam energy distribution ratio can be customized as expected. Both the simulation and test results verify the effectiveness of the proposed method. This method provides a new design idea for metasurface devices for multi-beam energy management. The meta-device has potential applications in satellite communications, holography, biomolecular detection, and radar systems. This device can also enhance the capacity of wireless communication.

## 1. Introduction

**Figure 1:**
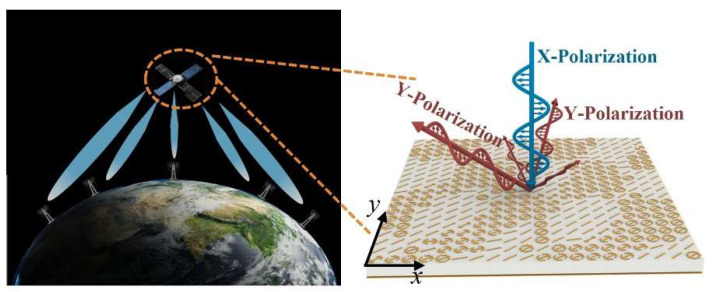
Multi-beam energy management customized meta-device for on-board transponders.

As human living environments become more diverse, high-quality ground-based communication is also facing challenges. This is particularly evident in environments with intricate ground conditions, such as dense forests, mountainous regions crisscrossed by gullies, desert and Gobi areas with sparse communication base stations, and buildings plagued by severe electromagnetic interference. Even the complex sea clutter in distant oceans stands as a critical factor impacting communications. Ground-based communication systems [1,2,3] typically adopt high-power, full-space coverage methods to ensure communication distance and range, often at the cost of increased weight and volume. Satellite communications [4,5], however, relay and retransmit electromagnetic (EM) waves emitted from the ground through space via on-board transponders, addressing communication challenges in complex environments to a certain extent. As shown in Figure 1, satellites can communicate with multiple ground base stations simultaneously, addressing the challenges posed by the distance between ground-based communication systems. For on-board satellite equipment, however, the payload is the primary consideration, imposing far stricter requirements and constraints on the weight and volume of on-board transponders compared with ground-based transmitters. Consequently, the beams of satellite communication systems must account for multi-directional beam generation and the attendant energy management issues tailored to communication targets [6,7]. Metasurfaces [8,9,10,11,12]—artificial electromagnetic structures—boast both low-profile and lightweight characteristics and can achieve exceptional electromagnetic wave manipulation. They thus provide an effective platform for addressing multi-beam directional shaping and energy management in satellite communication systems [13,14,15].

In traditional approaches, the manipulation of multi-beam EM waves based on metasurfaces often relies on empirical practices or brute-force optimization to determine the required phase and amplitude distribution of the metasurface [16,17,18]. These methods suffer from inherent drawbacks, including a low design accuracy and slow optimization process, while failing to effectively achieve precise beam energy allocation or concurrently constrain sidelobe levels. With the escalating demands for intelligence and low power consumption in communication systems, higher requirements are being placed on the efficiency of multi-beam energy management and the precision of beam position control. Traditional methods are increasingly ill-suited to this new trend in terms of time and energy costs, creating an urgent need for novel, rapid design schemes for customized multi-beam devices. Since the generated multi-beams can be expressed in a spherical coordinate system and projected onto a 2D plane consisting of elevation and azimuth angles, the ideal multi-beams correspond to the desired 2D wavefront [19,20]. This 2D wavefront can be quantitatively characterized as a desired 2D grayscale image through grayscale processing. In practical design, however, there exists a discrepancy between the actual 2D grayscale image corresponding to the multi-beams realized via metasurfaces and the desired 2D grayscale image. This insight inspired the optimization of multi-beam generation meta-devices using image matching algorithms. Image matching algorithms prefer to find feature-similar images among two or more images based on specific computational rules, and are widely applied in computer vision, pattern recognition, remote sensing detection, and intelligent driving [21,22,23,24]. Accordingly, the actual 2D grayscale image corresponding to the array can be calculated using the mapping model from the array’s amplitude–phase distribution to the far-field wavefront. The difference between this actual image and the desired image is computed via 2D image matching algorithms, and this difference is employed as the fitness function for the optimization algorithm. This enables accurate and rapid optimization design without the need for additional computational processing.

This work proposes a new method for constructing fully customized multi-beam meta-devices driven by image matching algorithms. Through a rapid data-driven optimization process, it successfully achieves the precise specification of beam position, intensity, and sidelobe constraints. A fully customized five-beam meta-device with varying energy allocations, constructed with amplitude–phase tunable meta-atoms, is employed to validate the proposed method. The resulting achieved energy-tailored multi-beam performance is presented in Figure 1. The process involves first performing grayscale processing on the 2D image of the far-field wavefront of the metasurface array, then establishing an optimization design model for a multi-beam energy management meta-device based on 2D image matching algorithms. Ultimately, this realizes a direct correlation between the far-field wavefront effect and the array’s amplitude–phase distribution—i.e., a direct mapping of “device function–design parameters”. A prototype of the meta-device finally obtained through this method was fabricated and tested. The experimental results agree with expectations, thereby verifying the method’s effectiveness. The proposed design scheme breaks through the limitations of traditional far-field pattern description, establishes an image-based functional function and matching optimization system, and provides a novel design approach for far-field pattern shaping.

## 2. Principle and Methods

According to the literature [25,26], the mapping model from the amplitude–phase distribution of the metasurface array to the far-field wavefront of the array $\;{\tilde{F}}_{\mathrm{Array}}\left(\theta ,\varphi ,l,\alpha ,f\right)$ can be obtained as follows:

(1a) $$\;{\tilde{F}}_{\mathrm{Array}}\left(\theta ,\varphi ,l,\alpha ,f\right)=\left|\frac{1}{n^{2}}\sum_{i=1}^{n}\sum_{j=1}^{n}\left|S_{11\mathrm{Cell}}^{⊥}\left(l_{i,j},\alpha_{i,j},f\right)\right|\;\exp\left(-j\Theta \right)\right|$$

(1b) $$\Theta =∠S_{11\mathrm{Cell}}^{⊥}\left(l_{i,j},\alpha_{i,j},f\right)+kp\left(j-0.5\right)\sin\theta \cos\varphi +kp(i-0.5)\sin\theta \sin\varphi$$

wherein k=2π/λ, *θ* and *φ* are the elevation angle and azimuth angle of the beam, respectively. $S_{11\mathrm{Cell}}^{⊥}\left(l_{i,j},\alpha_{i,j},f\right)$ is the orthogonally polarized reflection coefficient of the meta-atom, which is related to the meta-atom structural parameters, the fractal arm length *l,* and the rotation angle *α*. *i* and *j* denote the *i*-th row and *j*-th column of the meta-atom in the metasurface array. *p* is the period of the meta-atom. The detailed derivation process of the above formula refers to Section A of the Supplementary Materials (SMs). The above formula indicates that the far-field wavefront model of the multi-beam energy management device is closely related to frequency. In this work, as a verification of the proposed method, only the far-field wavefront at the central frequency of 10 GHz is considered, and the frequency term will be simplified in the subsequent model.

Next, starting from the function of the meta-device for multi-beam energy management, the analytical expression of the device’s function “*g*” is driven, and we establish the mapping relationship between the array amplitude–phase distribution and the function *g*, thereby guiding the rapid optimization design of the device.

According to the analytical expression principle of the function, we can concretely express the grayscale mappings *ξ* and *ζ* (from the amplitude and phase distribution of the array to the device actual function *g*, respectively) in the optimization design paradigm model of the amplitude–phase modulation metasurface as follows:

(2) $$\begin{array}{c} g\left(l,\alpha \right)=\left(\xi ⊕\zeta \right)\left[{\tilde{F}}_{\mathrm{Array}}\left(\theta ,\varphi ,l,\alpha \right)\right]\\ =255\left(\begin{array}{cccc} {\tilde{F}}_{\mathrm{Array}}\left(\theta_{1},\varphi_{1}\right) & {\tilde{F}}_{\mathrm{Array}}\left(\theta_{1},\varphi_{2}\right) & \ldots & {\tilde{F}}_{\mathrm{Array}}\left(\theta_{1},\varphi_{n}\right)\\ {\tilde{F}}_{\mathrm{Array}}\left(\theta_{2},\varphi_{1}\right) & {\tilde{F}}_{\mathrm{Array}}\left(\theta_{2},\varphi_{2}\right) & \ldots & {\tilde{F}}_{\mathrm{Array}}\left(\theta_{2},\varphi_{n}\right)\\ \vdots & \vdots & \ddots & \vdots \\ {\tilde{F}}_{\mathrm{Array}}\left(\theta_{m},\varphi_{1}\right) & {\tilde{F}}_{\mathrm{Array}}\left(\theta_{m},\varphi_{2}\right) & \ldots & {\tilde{F}}_{\mathrm{Array}}\left(\theta_{m},\varphi_{n}\right) \end{array}\right) \end{array}$$

For brevity, the meta-atom structure dimensions *l* and *α* are omitted from the matrix representation of the above formula. Now, the two-dimensional grayscale processing of the device function in the optimization and design model has been completed.

In satellite communication systems, to maximize energy efficiency, on-board transponders should generate ideal beams directed at the communication targets. These energy beams are expressed in a spherical coordinate system and projected onto a 2D plane consisting of elevation angles and azimuth angles to form the desired 2D wavefront. To quantitatively characterize this 2D wavefront, we perform grayscale processing on the 2D projection and take a step size of 1° in both the elevation angle and azimuth angle dimensions to generate a 90 × 359 2D image. The above processing process constitutes the following analytical expression of the expected function $\tilde{g}$ of the device:

(3) $$\begin{array}{c} \tilde{g}=\left(\begin{array}{cccc} h\left(\theta_{1},\varphi_{1}\right) & h\left(\theta_{1},\varphi_{2}\right) & \ldots & h\left(\theta_{1},\varphi_{n}\right)\\ h\left(\theta_{2},\varphi_{1}\right) & h\left(\theta_{2},\varphi_{2}\right) & \ldots & h\left(\theta_{2},\varphi_{n}\right)\\ \vdots & \vdots & \ddots & \vdots \\ h\left(\theta_{m},\varphi_{1}\right) & h\left(\theta_{m},\varphi_{2}\right) & \ldots & h\left(\theta_{m},\varphi_{n}\right) \end{array}\right)\\ \left\{\begin{array}{c} m=90\\ n=359\\ h\in \left[0,255\right] \end{array}\right. \end{array}$$

where $h\left(\theta_{1},\varphi_{1}\right)$ means the gray value *h* in direction of $\left(\theta_{m},\varphi_{n}\right)$.

Using binarized grayscale images to characterize the desired spatial multi-beams can more accurately describe the distribution position and intensity of spatial beams, while taking into account the constraints on sidelobe levels in space. In the process of optimizing the array using the heuristic optimization algorithm, the actual 2D grayscale image corresponding to the array can also be calculated based on the mapping model from the array’s amplitude–phase distribution to the far-field wavefront. The difference between this actual image and the expected image is computed via the 2D image matching algorithm, and this difference is used as the fitness function of the optimization algorithm. This enables accurate and rapid optimization design without additional computational processing.

Since the objective of this work is to achieve the controllable energy distribution of multi-beams, it is necessary to comprehensively consider the number of beams and their energy distribution, making this a multi-objective task. Existing optimization algorithms tend to conduct local searches during global optimization and thus fall into local optima. In contrast, image matching algorithms focus more on feature correspondence and global matching performance, thereby possessing advantages in energy-tailored multi-beam generation. Additionally, the computational efficiency of 2D image matching algorithms is more stable, and their performance is controllably affected by data scale. However, the computational efficiency of existing optimization techniques decreases significantly with the increase in parameter dimensions and the number of iterations. Considering the above factors comprehensively, the image matching algorithm is selected for the customized design of multi-beams.

In this work, considering the computation time and scale, the Mean Absolute Differences (MAD) algorithm [27] is selected as the image matching algorithm for array optimization. According to the principle of the image matching algorithm, the index calculation method of the MAD algorithm is an image similarity measurement function, also the objective function of the MAD algorithm, as follows [28]:

(4) $$D_{\mathrm{MAD}}=\frac{1}{mn}\sum_{i=1}^{m}\sum_{j=1}^{n}\left|g_{ij}-{\tilde{g}}_{ij}\right|$$

wherein *g* represents the actual 2D grayscale image calculated from the array amplitude–phase distribution and $\tilde{g}$ represents the expected image. The optimization model using the genetic algorithm is employed to perform specific optimization on the array. We take *D*_MAD_ as the fitness function of the genetic algorithm. To ensure that the far-field wavefront formed by the array achieves the expected effect, the number of array elements is minimized to realize rapid optimization design. Therefore, a 20 × 20 element array is adopted in this section. Each element contains two variable parameters: the fractal arm length *l* and the rotation angle *α*. Thus, the number of individual DNA loci in the model to be optimized by the genetic algorithm is given by

(5) $$N_{\mathrm{DNA}}=20\times 20\times 2=800$$

while other parameters of the genetic algorithm are set as follows:

(6) $$\left\{\begin{array}{l} N_{ini}=200\\ \varepsilon_{G}=10^{-4}\\ N_{\mathrm{GS}}=50\\ N_{G\max}=500\\ p_{C}=0.3\\ p_{M}=0.01 \end{array}\right.$$

The value of the objective function—namely the mean absolute difference—becomes smaller as the images become more similar. Therefore, the matching criterion and the constraint conditions of the optimization design model are finally established as follows:

(7) $$\begin{array}{c} \min f_{\mathrm{Adapt}}=D_{\mathrm{MAD}}=\frac{1}{mn}\sum_{i=1}^{m}\sum_{j=1}^{n}\left|g_{ij}\left(l,\alpha \right)-{\tilde{g}}_{ij}\right|\\ s.t.\left\{\begin{array}{l} m=90\\ n=359\\ h\in \left[0,255\right]\\ \alpha \in \left[10,85\right]\\ l\in \left[1,6\right] \end{array}\right. \end{array}$$

Here, as an example, we randomly select five directions (25, −45), (20, 20), (−30, −50), (−25, 30), (0, 0) to generate five beams with random energy allocations (40%, 20%, 20%, 10%, 10%). Then the desired function in Formula (3) can be written with the special value conditions in the desired function as follows:

(8) $$\left\{\begin{array}{l} h\left(25,-45\right)=0.4\times 255=102\\ h\left(20,20\right)=0.2\times 255=51\\ h\left(-30,-50\right)=0.2\times 255=51\\ h\left(-25,30\right)=0.1\times 255=25.5\\ h\left(0,0\right)=0.1\times 255=25.5\\ h\left(\theta_{\mathrm{other}},\varphi_{\mathrm{other}}\right)=0 \end{array}\right.$$

wherein the 2D grayscale values of the desired function for the five directions are consistent with the expected energy ratios.

**Figure 2:**
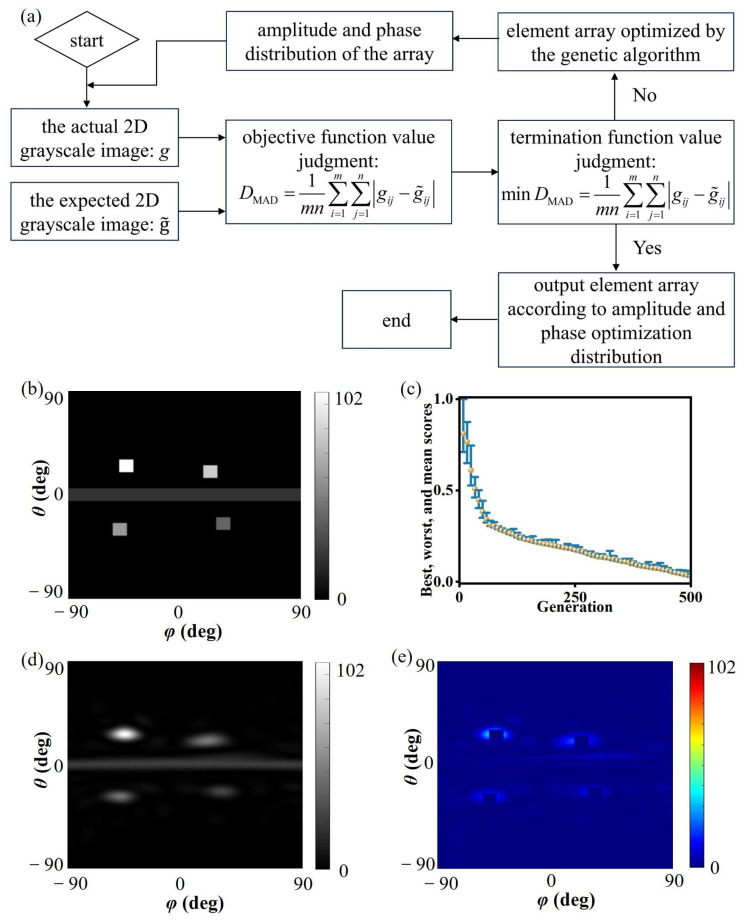
The performance of the optimization algorithm. (**a**) Flowchart of the optimization process. (**b**) Desired grayscale image; (**c**) genetic algorithm optimization design process, The blue bars represent the range between the optimal and worst values of each generation, and the yellow dots denote the average values. (**d**) grayscale image of genetic algorithm optimization result; (**e**) difference between desired and optimized grayscale images.

To present the algorithm’s process more clearly, a flow chart is provided in Figure 2a. The corresponding desired grayscale image is shown in Figure 2b. The desired grayscale image was substituted into the genetic algorithm optimization model [29,30,31], and the programming implementation was carried out in MATLAB (R2022a), with the optimization process shown in Figure 2c. After 495 rounds of survival competition, the change rate of the fitness function was less than the convergence criterion, indicating that the individual had reached the optimal state, i.e., the overall amplitude and phase optimization distribution process for the metasurface array had been completed. The grayscale image of the resulting optimized individual is shown in Figure 2d. Comparing the desired and actual grayscale images, the difference between them is shown in Figure 2e. The two are in good agreement, with only some errors in the main beam width range. This is because in the algorithm design process, strict restrictions were imposed on the beam width to ensure the accuracy of the designed beam. Thus, even if the beam generated by the actual array becomes wider, it can still be within the allowable range of the desired beam error. The above simulation process verifies the effectiveness of the genetic algorithm optimization.

## 3. Results

After theoretically verifying the accuracy of the proposed two-dimensional grayscale image matching algorithm, the next step is to construct a metasurface based on the optimization results to verify its actual effect, thereby further proving the practicability of the optimization method proposed in this work. The application requirements of the multi-beam energy management meta-device have weakened the energy constraints on co-polarized waves and cross-polarized waves, but increased the requirements for decoupling amplitude and phase regulation. Firstly, the mechanism of the independent regulation of amplitude and phase by cross-polarization conversion meta-atoms is analyzed based on the Jones matrix—details are shown in Section B of the Supplementary Materials. The results are as follows:

(9) $$r_{xy}=r_{yx}=e^{j\phi_{x}}\sin2\alpha$$

wherein *r* represents the reflection coefficient and the subscript *xy* denotes the *x*-polarized component (i.e., the cross-polarized component) reflected or transmitted by the incident y-polarized wave, and the meanings of other subscripts are similar to this. *Φ_x_* represents the reflection phase response of the structure to *x*-polarized waves in the initial state, while Formula (9) shows that the rotating angle of the metallic structure α can realize the scaling control of the cross-polarized wave amplitude from 0 to 100% when it changes from 0 to 45°. That is, it achieves full-range coverage of the cross-polarized wave amplitude regulation. For the reflection phase, the response of the structure to *x* and *y* linearly polarized EM waves is illumination, which are *Φ_x_* and *Φ_y_,* respectively, if they meet $\phi_{x}=\phi_{y}+180{}^{\circ}$ [12]; it is proved in Section B of the Supplementary Materials that the reflection phase of the cross-polarized wave is only related to the transmission phase *Φ_x_* of the structure under *x*-polarized wave illumination. Then, 360° full-range regulation of the reflection phase of the cross-polarized wave can be achieved by adjusting the transmission phase *Φ_x_* under *x*-polarization.

It is worth noting that in Formula (9), the amplitude scaling factor of the cross-polarization response is sine, so this factor is symmetric about *α* = 0°. That is, when -*α* is taken, it is equivalent to adding a 180° phase to the transmission phase *Φ_x_*. Therefore, in the meta-atom design, it is only necessary to construct a meta-atom whose transmission phase under *x-*polarization covers 180°, which greatly reduces the difficulty of meta-atom design. Next, a reflective amplitude–phase tunable meta-atom with decoupled amplitude and phase is designed based on the above mechanism [32,33]. The meta-atom is symmetric about both the *x*-axis and *y*-axis, make its transmission phase responses to *x*-polarized and *y*-polarized waves satisfy a 180° phase difference, and achieve the fully decoupled and complete regulation of the amplitude and phase of the cross-polarized reflected wave of the meta-atom by rotating the meta-atom and adjusting the transmission phase of the meta-atom under *x*-polarization to meet the 180° phase coverage. The details of the meta-atom are shown in Section C of the Supplementary Materials.

After selecting the amplitude–phase tunable meta-atom, parameter scanning simulations were performed to obtain the mapping relationship from structural parameters (*l*, *α*) to the amplitude $\Psi \left(l,\alpha ,f\right)$ and phase $\Phi \left(l,\alpha ,f\right)$ of the reflection coefficient. The concrete expression of the mappings G and H in the optimization design paradigm model of the amplitude–phase modulation metasurface established is completed as follows:

(10a) $$\Psi \left(l,\alpha ,f\right)=G\left[l,\alpha ,f\right]=\left|S_{11\mathrm{Cell}}^{⊥}\left(l,\alpha ,f\right)\right|$$

(10b) $$\Phi \left(l,\alpha ,f\right)=H\left[l,\alpha ,f\right]=∠S_{11\mathrm{Cell}}^{⊥}\left(l,\alpha ,f\right)$$

wherein G and H represent the process of obtaining the reflection coefficient through numerical simulation. Although both the amplitude and phase responses are related to *l* and *α* here, the phase is mainly affected by the branch length *l* and the amplitude is mainly affected by the rotation angle *α*. So far, the concrete expression of the mapping relationship at the meta-atom level in the optimization design paradigm model has been completed.

**Figure 3:**
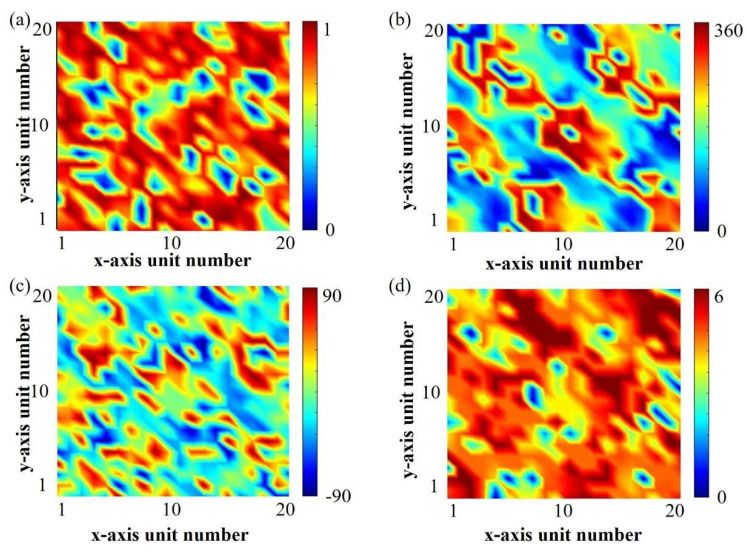
Performance distribution and corresponding meta-atom parameter distribution. (**a**) Amplitude distribution and (**b**) phase distribution required after genetic algorithm optimization; (**c**) rotation angle *α* and (**d**) fractal arm length *l* required for the corresponding amplitude–phase tunable meta-atom.

After decoding the optimal individual in previous steps, the optimized array amplitude–phase distribution is obtained as shown in Figure 3a,b. Then, combining the required amplitude–phase distribution with the amplitude–phase response mapping dataset generated earlier, as shown in Figure S2d,e of the Supplementary Materials, the structural parameters of the amplitude–phase tunable meta-atom designed in this section are obtained as shown in Figure 3c,d.

**Figure 4:**
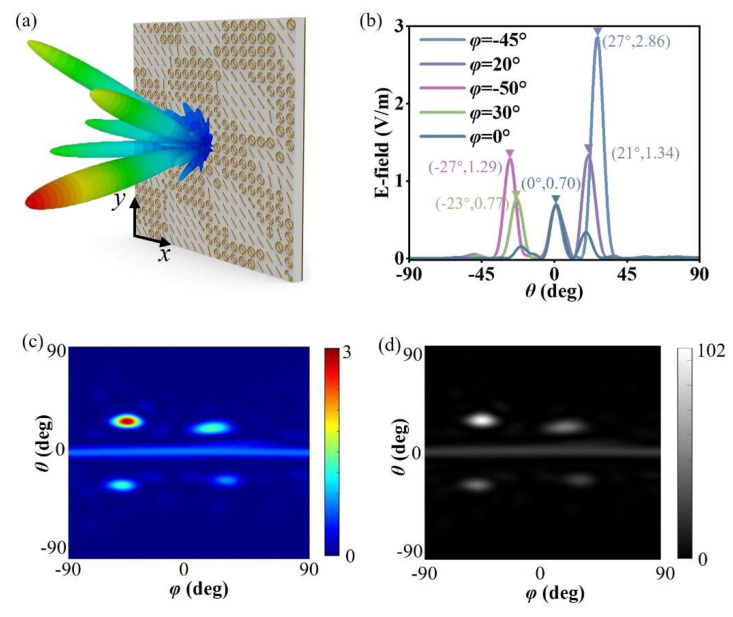
Array modeling and simulation results based on genetic algorithm optimization design. (**a**) Array modeling and 3D far-field pattern simulation results of genetic algorithm optimization design; (**b**) far-field patterns in the planes where each designed beam is located; (**c**) 2D far-field pattern simulation results; (**d**) 2D grayscale image corresponding to the simulation results.

Joint modeling and numerical simulation of the array were carried out using MATLAB and CST (v. 2020) [34], and the 3D far-field wavefront was obtained as shown in Figure 4a. As the metasurface array contains 20 × 20 unit cells, the size of the array is 200 mm × 200 mm. Meanwhile the boundaries are “open add space” in the Time Domain Solver of the CST. The x-polarized plane wave illuminates this metasurface normally. It can be seen from the figure that the metasurface device has multiple beams consistent with the expected directions and meeting the energy distribution conditions. To further show the direction and energy distribution of each beam, we plotted 1D wavefront curves in the azimuth plane where each main beam is located. It should be specially noted that since there may be multiple beams in the same azimuth plane, to avoid being misleading due to the display of far-field wavefronts of different beams, only the far-field wavefront curves in the quadrant where each main beam is located are plotted here, as shown in Figure 4b. The energy distribution ratio of each main beam is close to expectations, which again proves that the rapidly optimized metasurface device proposed in this section can realize the flexible regulation of the far-field wavefront of the reflected cross-polarized wave. In addition, the 3D far-field pattern is displayed as a 2D far-field pattern, as shown in Figure 4c, from which the five designed beams and their energy distribution can also be clearly seen. By comparing the expected 2D grayscale image in Figure 4a with the simulated 2D grayscale image in Figure 4d, it is found that the two are in good agreement, which verifies the effectiveness of the optimization design method for multi-beam energy management metasurfaces based on the 2D image matching algorithm proposed in this section.

**Figure 5:**
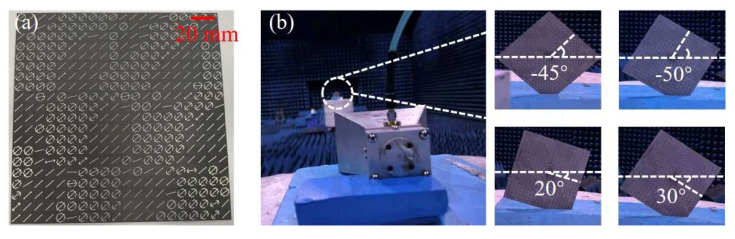
The photograph of the metasurface prototype and test setup. (**a**) Physical photograph of the multi-beam energy management metasurface prototype; (**b**) anechoic chamber test environment. Since the main beams are not located in the *xoz* or *yoz* plane, the prototype needs to be placed at an angle.

To further verify the practical effect of the multi-beam energy management metasurface designed in this section, the multi-beam customized meta-device is fabricated using the standard printed circuit board technology and tested in a microwave anechoic chamber. The physical printed circuit board is double-sided and copper-clad, with the designed pattern etched and retained on one side, and separated by an F4B (ε_r_ = 2.65, tanδ = 0.003) dielectric substrate in the middle. The top view of the fabricated sample is shown in Figure 5a and the total size is 200 mm × 200 mm (20 × 20 unit cells) with a total thickness of 1.572 mm (0.0524*λ*).

**Figure 6:**
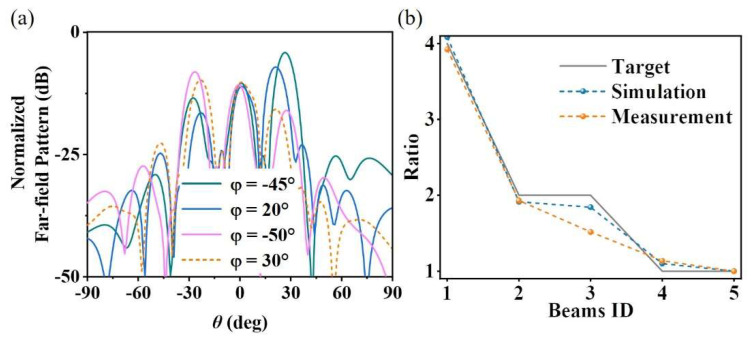
(**a**) Measured normalized RCS curves of the multi-beam energy management metasurface; (**b**) main beam energy ratios.

The actual measurement environment in the anechoic chamber is shown in Figure 5b. Double-ridged horn antennas are connected to both the input and output ports of the vector network analyzer (Agilent E8362C PNA, Santa Clara, CA, USA). The signal receiving horn is mounted on a fixed turntable, and the sample and signal transmitting horn are placed on a remote turntable that meets the far-field conditions. Since the design in this section is a cross-polarized reflective surface, the receiving horn and the transmitting horn are placed perpendicular to each other. To obtain a more accurate energy distribution of the main beams, the sample needs to be placed rotationally to ensure that the main beams are within the plane of the turntable. However, for the spherical coordinate system decomposition, the main beam at *θ* = 0 is the same for any *φ*, so there is no need to place the sample horizontally separately, and it is only necessary to place it at the four angles shown in Figure 5b. The far-field patterns in the section of each main beam obtained by far-field scanning are shown in Figure 6a. To intuitively show the peak energy ratio of each beam, we normalized the RCS. The experimental normalized RCS values of the five designed expected beams $\left(\theta_{m},\varphi_{n}\right)$ of (27, −45), (21, 20), (−27, −50), (−23, 30), (0, 0) are (−4.14, −7.07, −8.08, −9.73, −9.89), respectively. Figure 6b shows the main beam energy ratios under expected, simulated, and experimental conditions. It can be seen from the figure that the energy ratios between multiple main beams in the simulation and experiment are consistent with the design expectations, which verifies the effectiveness and accuracy of the optimization design method for multi-beam energy management metasurfaces proposed in this section. The efficiency of the designed device is calculated by integrating the 3dB energy bandwidth of the five main beams and integrating the energy of the reflected beam from an equal-sized metal plate. After calculation, the experimental efficiency is 71.7%.

To better demonstrate the excellent performance of the designed device, we compared its key performance metrics with multi-beam devices reported in the existing literature. The comparison results are presented in Table 1. These results indicate that the designed meta-device has advantages in terms of the number of beams and design efficiency, as well as the flexibility of the design process.

To conclude, the limitations of the image matching algorithm are discussed. For a greater number of beams and more precise energy ratios, greater computational load is demanded. Time complexity could quantify the computational load requirements. The algorithm exhibits a time complexity of O (NlogN + M × K), where N is the number of feature points in a single image, and M and K denote the number of feature points in two respective images. The number of feature points typically ranges from thousands to tens of thousands. Under more beams conditions, this complexity results in a surge in computational load. During the grayscale processing of the 2D projection, sampling is performed at a step size of 1° in both elevation and azimuth, generating a 90 × 359 2D image. Thus the resolution of the grayscale image is therefore 1°. Also, the impact of manufacturing errors is discussed. Manufacturing tolerances alter the reflective amplitude and phase characteristics of metasurface unit cells, thereby causing the beam direction and energy distribution ratio to deviate from the expected values of the optimal solution; notably, the reflective amplitude of metasurface unit cells is modulated by the rotation angle of the metal pattern, while the reflective phase is governed by the arm length of the I-shaped metal structure. In the optimal solution, the rotation angles of metasurface unit cells vary smoothly without abrupt fluctuations and the arm length values are concentrated, rendering the impact of arm length variations negligible; a comprehensive analysis confirms that this structural design exhibits low sensitivity to manufacturing errors.

## 4. Conclusions

This paper integrates a metasurface design with a two-dimensional image matching algorithm from the field of image processing and establishes a metasurface beam full-customization model that combines beam direction customization and beam energy management. Binary grayscale images are used to characterize the actual multi-beams and the expected spatial multi-beams. An image matching algorithm is employed to calculate the difference between the two, which serves as the basis for array optimization. Finally, a metasurface device capable of generating customized beam directions and energy distributions is obtained. The optimization design method for metasurfaces with multi-beam energy management proposed in this work provides a new design idea for far-field pattern shaping.

## Figures and Tables

**Table 1:** Comparison of characteristics between meta-device with others.

Ref	Beams	BSR (°)	EFF (%)	Layers	Band	Methods/ Flexibility	Speed	Accuracy (°)
[35]	4	34.1	70	1	6.31%	coding/low	>1 h	1
[36]	4/2	30	60.6	2	14.8%	parameters sweep/ low	>20 min	2
[37]	2	30	84.7	1	7.8/ 18.3 GHz	parameters sweep/ low	>40 min	1
[38]	4	30	none	1	13%	parameters sweep/ low	none	1
this work	5	30/20	71.7	1	10 GHz	data-driven optimization/high	5 min	1

BSR, Beam steering range; EFF, efficiency; ACC, accuracy; Layers, the layers of substrate; Band, bandwidth; Rapidity, the time spent on the device design process.

## Data Availability

The original contributions presented in this study are included in the article/Supplementary Materials. Further inquiries can be directed to the corresponding authors.

## Supplementary Materials

The following supporting information can be downloaded at: https://www.mdpi.com/article/10.3390/ma19010004/s1, Figure S1. Schematic diagram of far-field synthesis for the metasurface array. Figure S2. Design process of the reflective amplitude-phase tunable meta-atom.

## References

[1] Yu H., Lee H., Jeon H. (2017). What is 5G? Emerging 5G mobile services and network requirements. Sustainability.

[2] Wang J.H., Ling X.T., Le Y.W., Huang Y.M., You X.H. (2021). Blockchain-enabled wireless communications: A new paradigm towards 6G. Natl. Sci. Rev..

[3] Wu H., Shao R., Xu Z., Wu J.-W., Tan S., Wang X., Qi Z., Cheng Q., Zheng Y., Luo Y. (2025). A programmable metasurface antenna that approaches the wireless information mapping limit. Nat. Electron..

[4] Xi J., Suo Z., Ti J. (2025). The First experimental validation of a communication base station as a ground-based SAR for deformation monitoring. Remote Sens..

[5] Abdulkarim Y.I., Deng L., Awl H.N., Muhammadsharif F.F., Altintas O., Karaaslan M., Luo H. (2020). Design of a broadband coplanar waveguide-fed antenna incorporating organic solar cells with 100% Insolation for Ku band satellite communication. Materials.

[6] Tang X., Yan C., Sun H., Meng L., He Y., Liu R., Wang S., Wang L. (2025). A high-precision frequency synchronization method based on a novel geostationary communication satellite phase-locked transponder. Remote Sens..

[7] Kudsia C., Cameron R., Tang W.-C. (1992). Innovations in microwave filters and multiplexing networks for communications satellite systems. IEEE Trans. Microw. Theory Tech..

[8] Zhao P., Ding X., Li C., Tang S. (2023). Achieving photonic spin hall effect, spin-selective absorption, and beam deflection with a vanadium dioxide metasurface. Materials.

[9] Jiang H., Sheng L., Luo Y., Meng L., Cao W. (2023). Design of tunable broadband graphene-based metasurface with amplitude-phase modulation. Materials.

[10] Wang D., Cui X., Liu D., Zou X., Wang G., Zheng B., Cai T. (2024). Multi-characteristic integrated ultra-wideband frequency selective rasorber. Prog. Electromagn. Res..

[11] Cai T., Zhong Y., Liu D., Huang H., Wang D., Yang Y., Chen H., Lin X. (2024). Observation of polarization-maintaining near-field directionality. Prog. Electromagn. Res..

[12] Cui X., Liu D., Wang Z., Wang D., Wu B., Wang G., Zheng B., Cai T. (2023). Wideband and high-efficiency spin-locked achromatic meta-device. Nanophotonics.

[13] Wang C., Xu H.-X., Hu G., Liu Y., Liu T., Wang K., Zhang F., Xu S., Xu J., Pang Z. (2023). Full-space spin-decoupled versatile wavefront manipulations using non-interleaved metasurface. Nanophotonics.

[14] Macro D.R., Alessio Z., Merouane D., Mohamed S.A., Yuen C., Rosny J., Tretyakov S. (2020). Smart radio environments empowered by reconfigurable intelligent surfaces: How it works, state of research, and road ahead. IEEE J. Sel. Areas. Commun..

[15] Sievenpiper D.F., Schaffner J.H., Song H.J., Loo R.Y., Tangonan G. (2003). Two-dimensional beam steering using an electrically tunable impedance surface. IEEE Trans. Antennas Propag..

[16] Bai X., Tan S., Mikki S., Li E., Cui T.-J. (2024). Information-theoretic measures for reconfigurable metasurface-enabled direct digital modulation systems: An electromagnetic perspective. Prog. Electromagn. Res..

[17] Liang J.-C., Zhang L., Luo Z., Jiang R.-Z., Zhang W.-C., Wang S.-R., Sun M.-K., Jin S., Cheng Q., Cui T.-J. (2024). A filtering reconfigurable intelligent surface for interference-free wireless communications. Nat. Commun..

[18] Zhang M., Yao S., Zhu B., Lin J., Tao J., Shang W., Zhang W. (2024). Movable typography inspired tunable meta-cylinder for multi-directional microwave beam shaping. Adv. Mater. Technol..

[19] Zhuo Y., Mao T., Li H., Sun C., Wang Z., Han Z., Chen S. (2024). Multi-beam integrated sensing and communication: State-of-the-art, challenges and opportunities. IEEE Commun. Mag..

[20] Shen D., Dai L., Su X., Suo S. (2023). Multi-beam design for near-field extremely large-scale RIS-aided wireless communications. IEEE Trans. Green Commun. Netw..

[21] Zhu H., Zhao Y.G. (1999). Fast image correlative matching based on genetic algorithm. J. Infrared Millim. Waves.

[22] Yue G., Xu Y. An area based on image matching algorithm and its implementation. Proceedings of the 2012 Third World Congress on Software Engineering.

[23] Zhang X., Feng Z. New development of the image matching algorithm. Proceedings of the Ninth International Conference on Graphic and Image Processing (ICGIP 2017).

[24] Kazmer D.O., Olanrewaju R.H., Elbert D.C., Nguyen T.D. (2024). Design of shape forming elements for architected composites via bayesian optimization and genetic algorithms: A concept evaluation. Materials.

[25] Wu B., Liu T., Wang G., Cui X., Jia Y., Wang Y., Zhai H. (2025). Integrated electromagnetic sensing system based on a deep-neural-network-intervened genetic algorithm. Photon. Res..

[26] Zhang H., Zhang L., Liu S., Mao Z., Ma Y., He P.-H., Cui W.-Y., Huang Y.-F., Yang Q., Cui T.-J. (2024). Measurement of time-range-angle-dependent beam patterns of frequency diverse arrays. Prog. Electromagn. Res..

[27] Feng N., Wang H., Wang X., Zhang Y., Qian C., Huang Z., Chen H. (2024). Highly accurate and efficient 3D implementations empowered by deep neural network for 2DLMs-based metamaterials. Prog. Electromagn. Res..

[28] Cui X., Wang G., Wang D., Li X., Cai T., Liu K. (2021). Ultra-broadband transmissive gradient metasurface based on the topologically coding optimization method. Opt. Express.

[29] Chen M., Shen L., Hua Y., Qin Z., Wang H. (2024). Topology-Optimized Plasmonic Nanoantenna for Efficient Single-Photon Extraction. Prog. Electromagn. Res..

[30] Youssef A., Paola C., Tania P., Pier P. (2024). Smartphone-Integrated YOLOv4-CNN Approach for Rapid and Accurate Point-of-Care Colorimetric Antioxidant Testing in Saliva. Prog. Electromagn. Res..

[31] Fan J.A. (2020). Freeform metasurface design based on topology optimization. MRS Bull..

[32] Mounia D., Mohamed L., Julien S., Massimiliano C. (2025). Efficient Design of a Novel Multibeam Antenna Using Scalar Metasurfaces. Prog. Electromagn. Res..

[33] Saiful I., Van L., Tae H., Hyoungsuk Y. (2024). Wave Manipulation with mmWave Wide Bandwidth and Extensive Spatial Coverage Using 1-Bit Reconfigurable Intelligent Surface. Prog. Electromagn. Res..

[34] Tan Q., Qian C., Chen H. (2023). Inverse-Designed Metamaterials for on-Chip Combinational Optical Logic Circuit. Prog. Electromagn. Res..

[35] Li S., Han B., Li Z., Liu X., Huang G., Li R., Cao X. (2022). Transmissive coding metasurface with dual-circularly polarized multi-beam. Opt. Express.

[36] Guo W., Yuan F., Xu H. (2024). Broadband generation of a multi-polarization multi-beam using a receiving-transmitting metasurface. Opt. Express.

[37] Huang H., Liu F. (2025). Spin Decoupling-Scalar Holographic Impedance Hybrid Metasurface for Bidirectional Multibeams. Prog. Electromagn. Res. Lett..

[38] Ma X., Chen S., Han J., Mu Y., Liu H., Li L. (2024). Bidirectional multi-beam with multi-polarizations tensor holographic metasurface using bilayer anisotropic elements. Opt. Express.

