# Supplementary material for: Image Matching Algorithm-Driven Multi-Beam Customized Meta-Device
Source: Materials (Basel). 2025 Dec 19;19(1):4. doi: 10.3390/ma19010004 (PMC12786977; doi:10.3390/ma19010004)
Supplement: Supplementary file 1 [file materials-19-00004-s001.zip › materials-4002154-supplementary.pdf]

materials

MDPI

Supplementary Materials

Image Matching Algorithm-Driven Multi-Beam Customized Meta-Device

Xingshuo Cui [1,†], Dan Liu [2,†], Borui Wu [3,*,†], Huiyong Zeng [1,*], Yueyi Qin [1], Guangzheng Ren [1], Guangming Wang [1] and Tong Cai [1,*]

1 Air and Missile Defense College, Air Force Engineering University, Xi'an 710051, China; cxsgfs521@sina.com (X.C.); qinyy010404@163.com (Y.Q.); guangzheng_ren@163.com (G.R.); wgming01@sina.com (G.W.)
2 State Key Laboratory of Modern Optical Instrumentation, The Electromagnetics Academy Zhejiang University, Hangzhou 310027, China; 12231114@zju.edu.cn
3 Air Traffic Control and Navigation School, Air Force Engineering University, Xi'an 710051, China
* Correspondence: wuberry1118@163.com (B.W.); hyzeng.1023@163.com (H.Z.); caitong326@sina.cn (T.C.)
† These authors contributed equally to this work.

Section A: Derivation of Multi-beam Formation Mechanism

Regarding the principle of multi-beam formation, an analogy can be drawn from the perspective of antenna array beamforming. A certain number of antennas arranged in a specific pattern form an antenna array. Similarly, a metasurface array is composed of a certain number of units arranged in accordance with a specific amplitude–phase gradient variation rule. Therefore, the electromagnetic response of a metasurface array can be analogous to that of an antenna array.

Figure S1 provides an intuitive analogy between the antenna array and the metasurface array. Below, taking the reflective metasurface as an example, the derivation of the electromagnetic response model for the coded metasurface array will be carried out.

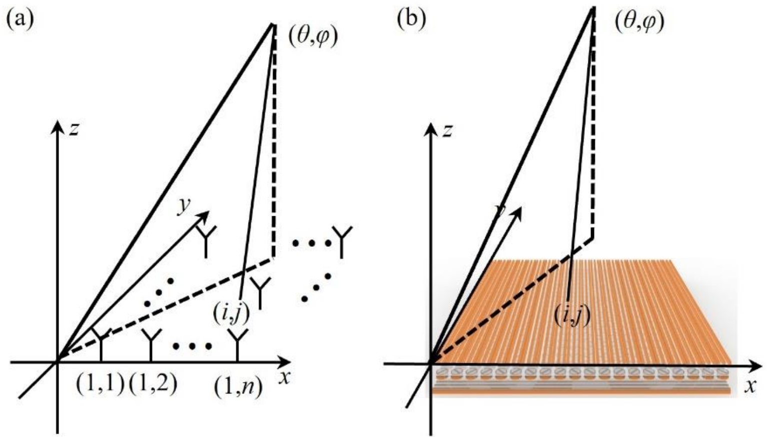

Figure S1. (a) Antenna array; (b) schematic diagram of far-field synthesis for the metasurface array.

According to the superposition theorem of electric fields, the electromagnetic response model of each unit in the array is regarded as the pattern function of each element in the array, and the arrangement relationship between units is regarded as the array factor in the array. Based on the pattern multiplication principle, the electric field distribution of the array in the far-field can be obtained by multiplying the element pattern function with the array factor, as shown below:

$$\vec{\boldsymbol{E}}_{Array}\left(\theta,\varphi\right)=\sum_{i=1}^{n}\sum_{j=1}^{n}\vec{\boldsymbol{E}}_{i,j}\left(\theta,\varphi\right)=K_{i,j}\,\boldsymbol{f}_{i,j}\left(\theta,\varphi\right)\frac{\exp\left(jkr_{i,j}\right)}{r_{i,j}} \tag{S1}$$

Among them, $K_{i,j}$ is the superposition proportional coefficient, $\boldsymbol{f}_{i,j}$ represents the radiation pattern coefficient of the unit, and $r_{i,j}$ represents the distance between the observation point and the unit. When the distance of the observation point satisfies the far-field condition, the characteristics of the unit can be ignored, that is, $K_{i,j}$ and $\boldsymbol{f}_{i,j}$ are neglected. When the feed source of the metasurface array is set as a normally incident TEM plane wave, the above formula can be expressed in the form of a far-field pattern function.

$$F_{\text{Array}}\left(\theta,\varphi,f\right)=\sum_{i=1}^{n}\sum_{j=1}^{n}\text{Amp}_{i,j}\exp\left(\Psi\right) \tag{S2a}$$

$$\Psi=\left[-j\left(Pha_{i,j}+kp\left(j-0.5\right)\sin\theta\cos\varphi+kp(i-0.5)\sin\theta\sin\varphi\right)\right] \tag{S2b}$$

In the above formula, $k = 2\pi/\lambda$ is the wave number of the incident plane wave and p is the periodic length of the meta-unit. In the main text, for the convenience of calculation in the image matching algorithm, the far-field pattern response model of the metasurface array is normalized and its modulus is taken, resulting in Equation 1 in the main text.

Section B: Amplitude–Phase Independent Regulation Mechanism

The mechanism of the independent regulation of amplitude and phase by a cross-polarization conversion meta-atom is analyzed based on the Jones matrix. For the meta-

atom, under the illumination of linearly polarized waves, its electromagnetic response can be expressed by the Jones matrix as follows:

$$\boldsymbol{R} = \begin{pmatrix} r_{xx} & r_{yx} \\ r_{xy} & r_{yy} \end{pmatrix} \tag{S3a}$$

$$\boldsymbol{T} = \begin{pmatrix} t_{xx} & t_{yx} \\ t_{xy} & t_{yy} \end{pmatrix} \tag{S3b}$$

wherein r and t represent the reflection coefficient and transmission coefficient, respectively. The subscript xy denotes the x-polarized component (i.e., cross-polarized component) reflected or transmitted by the incident y-polarized wave, and the meanings of other subscripts are similar to this. Moreover, the Jones matrices in the reflection system and transmission system have the same form. For the sake of simplicity in expression, only the mechanism analysis under the reflection system will be presented in the subsequent text, and the transmission system is completely the same. Usually, for the convenience and accuracy of design and analysis, a double-symmetric structure is mostly adopted, that is, the structure is symmetric about both the x-axis and y-axis. In this case, the electromagnetic response of the structure to the incident linearly polarized wave remains co-polarized without generating cross-polarized components, so the Jones matrix can be simplified as follows:

$$\boldsymbol{R} = \begin{pmatrix} r_{xx} & 0 \\ 0 & r_{yy} \end{pmatrix} \tag{S4}$$

At this point, the structure is rotated counterclockwise by an angle α along its geometric center, forming a rotation matrix as follows:

$$\boldsymbol{P}(\alpha) = \begin{pmatrix} \cos\alpha & \sin\alpha \\ -\sin\alpha & \cos\alpha \end{pmatrix} \tag{S5}$$

Then the rotated Jones matrix is given by

$$\boldsymbol{R}'(\alpha) = \boldsymbol{P}^{-1}(\alpha)\boldsymbol{R}\boldsymbol{P}(\alpha) = \begin{pmatrix} r_{xx}\cos^2\alpha + r_{yy}\sin^2\alpha & \frac{1}{2}\left(r_{xx} - r_{yy}\right)\sin 2\alpha \\ \frac{1}{2}\left(r_{xx} - r_{yy}\right)\sin 2\alpha & r_{xx}\cos^2\alpha + r_{yy}\sin^2\alpha \end{pmatrix} \tag{S6}$$

It can be seen from the above formula that the rotation angle α of the structure acts as a scaling factor in the amplitude of the cross-polarized wave. Due to the periodicity of the sine function of double angles, theoretically, rotating the structure within the range of 0-45° can realize the scaling control of the cross-polarized wave amplitude from 0 to 100%, that is, achieve the full-range coverage of the cross-polarized wave amplitude regulation.

Next, the phase regulation method of the cross-polarized wave will be discussed. The electromagnetic response in the above formula is written in exponential form as follows:

$$\boldsymbol{R}'(\alpha) = \begin{pmatrix} \mathrm{e}^{j\phi_x}\cos^2\alpha + \mathrm{e}^{j\phi_y}\sin^2\alpha & \frac{1}{2}\left(\mathrm{e}^{j\phi_x} - \mathrm{e}^{j\phi_y}\right)\sin 2\alpha \\ \frac{1}{2}\left(\mathrm{e}^{j\phi_x} - \mathrm{e}^{j\phi_y}\right)\sin 2\alpha & \mathrm{e}^{j\phi_x}\cos^2\alpha + \mathrm{e}^{j\phi_y}\sin^2\alpha \end{pmatrix} \tag{S7}$$

wherein Φ_x and Φ_y, respectively, represent the reflection phase response of the structure to x and y linearly polarized waves in the initial state, also known as the transmission phase of the structure. Their values are only related to the structural dimensions, which means that when the structural dimensions are determined, Φ_x and Φ_y are already fixed. Here, we may assume $\phi_x = \phi_y + 180°$, then the reflection coefficient in the above formula can be simplified as

$$r_{xx} = r_{yy} = \mathrm{e}^{j\phi_x}\cos 2\alpha \tag{S8a}$$

$$r_{xy} = r_{yx} = \mathrm{e}^{j\phi_x}\sin 2\alpha \tag{S8b}$$

The above formula indicates that the reflection phase of the cross-polarized wave is only related to the transmission phase Φ_x of the structure under x-polarized wave illumination. According to the fixed relationship between the transmission phase Φ_y and Φ_x, as long as the meta-atom satisfies a 180° phase difference between x- and y-polarizations, 360° full-range regulation of the reflection phase of the cross-polarized wave can be achieved by adjusting the transmission phase under x-polarization. It is worth noting that in Formula (S8b), the amplitude scaling factor of the cross-polarization response is a sine function, so this factor is symmetric about α = 0°. That is, when -α is taken, it is equivalent to adding a 180° phase to the transmission phase Φ_x.

Section C: Analysis of Meta-atom Configuration

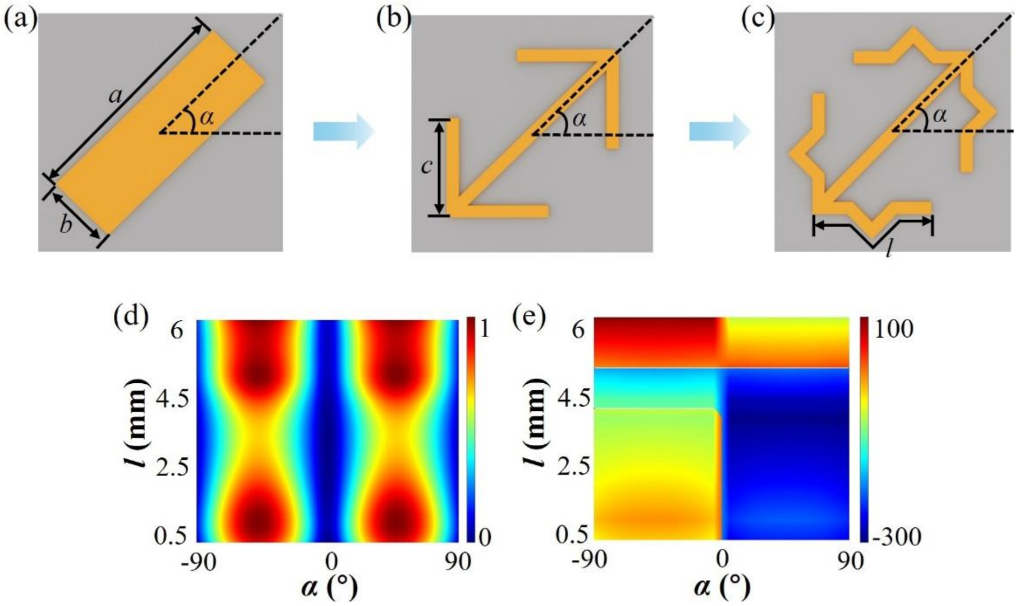

Figure S2. Design process of the reflective amplitude–phase tunable meta-atom. (a) Initial rectangular patch structure; (b) adding right-angle arms to improve polarization conversion efficiency; (c) adjusting to a first-order Koch curve arm structure to expand the phase regulation range; (d) amplitude regulation range of the final fractal arm amplitude–phase tunable meta-atom; (e) phase regulation range of the final fractal arm amplitude–phase tunable meta-atom.

A reflective amplitude–phase tunable meta-atom with decoupled amplitude and phase is designed based on the above mechanism. As shown in Fig. S2(a), the unit consists of a 1.5 mm-thick F4B dielectric substrate (ε_r = 2.65, tanδ = 0.003), an upper layer copper ($\sigma = 5.8\times10^7$ S/m) metal pattern, and a bottom copper metal ground plane. The copper has a thickness of 0.036 mm. The unit period is p = 10 mm, and the dimensions of the metal patch are a = 9 mm and b = 2.6 mm. In Figure S2(b) and (c), the length of the middle metal line in the I-shaped metal pattern is also a = 9 mm, with a metal line width of 0.5 mm. We first consider the co-polarized reflected wave transmission phase of the meta-atom under the independent incidence of x-polarized and y-polarized waves. By changing the length a and width b of the rectangular patch, it is made to satisfy a 180° phase difference between the reflection transmission phases under x-polarization and y-polarization. The flexible regulation of the amplitude of the cross-polarized wave can be achieved by rotating the rectangular patch counterclockwise along its geometric center, and the flexible regulation of the phase of the cross-polarized wave can be achieved by changing the dimension a of the rectangular patch along the x-direction. However, considering that the rectangular patch has a narrow resonant frequency, and the transmission phase of y-polarization does not change when adjusting the dimension a, it is difficult to maintain the 180° phase difference. By adding right-angle metal arms at both ends of the rectangular patch, parameters that can simultaneously regulate the transmission phases of x- and y-polarizations are introduced. The improved structure is shown in Fig. S2(b). By adjusting the length c of the

right-angle metal arms, the transmission phase of x-polarization can be regulated while maintaining a stable phase difference between x- and y-polarizations to a certain extent.

The above improvement method increases the cross-polarization conversion rate of the meta-atom for linearly polarized incident waves to a certain extent. However, since the length c of the metal arm is limited by the meta-atom period length p, it is difficult to achieve 180° coverage of the transmission phase of x linearly polarized waves by adjusting c, which often requires complex scanning simulation and the optimization of structural parameters. Therefore, improving the regulation capability of the structure on the transmission phase of x-polarized waves becomes the key to simplifying the design. Benefiting from the application of the self-similarity and plane-filling properties of fractal geometry in metasurfaces and antennas, an effective idea is provided to solve this problem. We further improved the meta-atom as shown in Fig. S2(c). Considering the modeling complexity and processing technology, this work only borrowed the form of the first-order Koch curve in fractal geometry to fold the right-angle metal arms into three equal parts. By changing l, a wide-range adjustment of the transmission phase of x-polarization can be achieved while maintaining a stable 180° difference from the transmission phase of y-polarization.

After selecting the amplitude–phase tunable meta-atom shown in Fig. S2(c) in this section, the independent and continuous regulation of the amplitude and phase of the reflected cross-polarized wave can be achieved by adjusting the fractal arm length l and the counterclockwise rotation angle α around the geometric center. Parameter scanning simulations are completed by sampling l continuously from 0.5 to 6 with a step size of 0.5, and sampling α continuously from 5° to 85° with a step size of 5°, as shown in Figs. S2(d)-(e). The results show that the orthogonally polarized reflection phase is mainly determined by l, and the reflection amplitude is mainly determined by α, which is consistent with the previous analysis. The simulation results also provide a basis for constructing the mapping between structural parameters and reflection amplitude–phase in the optimization model.
